# Investigating rectal toxicity associated dosimetric features with deformable accumulated rectal surface dose maps for cervical cancer radiotherapy

**DOI:** 10.1186/s13014-018-1068-0

**Published:** 2018-07-06

**Authors:** Jiawei Chen, Haibin Chen, Zichun Zhong, Zhuoyu Wang, Brian Hrycushko, Linghong Zhou, Steve Jiang, Kevin Albuquerque, Xuejun Gu, Xin Zhen

**Affiliations:** 1 School of Biomedical Engineering, Southern Medical University, Guangzhou, 510515 Guangdong China; 2 Department of Radiation Oncology, The University of Texas, Southwestern Medical Center, Dallas, TX 75390 USA; 3 Department of Computer Science, Wayne State University, Detroit, MI 48202 USA; 4 Department of Epidemiology, Biostatistics and Occupational Health, McGill University, 805 Sherbrooke Street West, Montreal, Quebec H3A 0G4 Canada

**Keywords:** Rectum toxicity prediction, Machine learning, Dose accumulation, Deformable registration, Cervical cancer

## Abstract

**Background:**

Better knowledge of the dose-toxicity relationship is essential for safe dose escalation to improve local control in cervical cancer radiotherapy. The conventional dose-toxicity model is based on the dose volume histogram, which is the parameter lacking spatial dose information. To overcome this limit, we explore a comprehensive rectal dose-toxicity model based on both dose volume histogram and dose map features for accurate radiation toxicity prediction.

**Methods:**

Forty-two cervical cancer patients treated with combined external beam radiotherapy (EBRT) and brachytherapy (BT) were retrospectively studied, including 12 with Grade ≥ 2 rectum toxicity and 30 patients with Grade 0–1 toxicity (non-toxicity patients). The cumulative equivalent 2-Gy rectal surface dose was deformably summed using the deformation vector fields obtained through a recent developed local topology preserved non-rigid point matching algorithm. The cumulative three-dimensional (3D) dose was flattened and mapped to a two-dimensional (2D) plane to obtain the rectum surface dose map (RSDM). The *dose volume parameters* (DVPs) were calculated from the 3D rectum surface, while the texture features and the *dose geometric parameters* (DGPs) were extracted from the 2D RSDM. Representative features further computed from DVPs, textures and DGPs by principle component analysis (PCA) and statistical analysis were respectively fed into a support vector machine equipped with a sequential feature selection procedure. The predictive powers of the representative features were compared with the GEC-ESTRO dosimetric parameters D_0.1/1/2cm_^3^.

**Results:**

Satisfactory predictive accuracy of sensitivity 74.75 and 84.75%, specificity 72.67 and 79.87%, and area under the receiver operating characteristic curve (AUC) 0.82 and 0.91 were respectively achieved by the PCA features and statistical significant features, which were superior to the D_0.1/1/2cm_^3^ (AUC 0.71). The relative area in dose levels of 64Gy, 67Gy, 68Gy, 87Gy, 88Gy and 89Gy, perimeters in dose levels of 89Gy, as well as two texture features were ranked as the important factors that were closely correlated with rectal toxicity.

**Conclusions:**

Our extensive experimental results have demonstrated the feasibility of the proposed scheme. A future large patient cohort study is still needed for model validation.

**Electronic supplementary material:**

The online version of this article (10.1186/s13014-018-1068-0) contains supplementary material, which is available to authorized users.

## Background

The combination of the external beam radiotherapy (EBRT) and brachytherapy (BT) (EBRT+BT) is a common therapy regime for locally advanced cervical cancer [1]. Recent monocentric and multicentric EBRT+BT studies [2–4] have shown promising results with high tumor local control rate. However, radiation induced side effect (or toxicity) on organs at risk (OARs), such as rectum, bladder and vagina, is still a concern. Serious side effects such as bowel obstruction can occur months to years after treatment and impact negatively on the patients’ quality-of-life. The correlation between OARs’ morbidity and radiation dose parameters was analyzed in EMBRACE study [2]. Particularly, the D_0.1cm_^3^, D_1cc_ and D_2cm_^3^ of rectum were used to establish dose-toxicity relationship in the occurrence of rectal morbidity. D_0.1/1/2cm_^3^ are conventional *dose volume parameters* (DVPs) extracted from dose volume histogram (DVH). Inherently, they are in deficiency of dosimetric spatial information. Studies have shown close relationship between the spatial dose characteristics and rectal toxicity [5–12]. For instance, Wortel et al. [12] observed significant differences in local rectal dose distribution between prostate cancer patients with and without toxicity by utilizing the unfolded two dimensional (2D) rectum surface dose map (RSDM). Similarly, Munbodh et al. [10] demonstrated that late rectal toxicity was related to dose on the upper rectum region by investigating dose pattern on the RSDM. Buettner et al. [6] analyzed the RSDM and found significant correlation between the subjective sphincter control and the dose delivered to the anal sphincter region. Another issue with current D_0.1/1/2cm_^3^ evaluation procedure is that the cumulative dose is summed with an assumption that the hotspot regions are stationary throughout the entire fractional treatments [13, 14]. However, this static assumption is often violated by the large inter-fraction rectum deformation, especially in intra-cavity brachytherapy treatment cases [15–17]. Recently, promising advancements have been reported by Moulton et al. who investigated the associations between RSDM and gastrointestinal toxicities after deformably registering each phase of a combined EBRT-BT prostate cancer treatment [18]. These limited but inspiring studies shed light on the possibility of revealing more accurate dose-toxicity relationship by exploring the spatial dose distribution patterns on the deformable accumulated dose.

In this study, we proposed and evaluated a rectum dose-toxicity prediction scheme using both dose volume parameters and dose map spatial information. In addition, the accumulated rectal dose maps are obtained with the aid of an accurate deformable image registration. The accumulated 3D rectal surface dose was flattened to obtain a 2D RSDM. The DVPs were extracted from the DVHs of cumulative dose, while the texture features and the *dose geometric parameters* (DGPs) were extracted from the 2D RSDM. Representative features further computed from DVPs, textures and DGPs by principle component analysis (PCA) and statistical analysis were respectively feed into a support vector machine (SVM) equipped with a sequential feature selection (SFS) procedure. The predictive powers of the representative features were compared with the GEC-ESTRO dosimetric parameters D_0.1/1/2cc_.

## Methods

### Patient cohort

Forty-two cervical cancer patients were retrospectively studied. These patients were treated with EBRT and BT. EBRT treatment plans were generated on the Pinnacle treatment planning system (Philips Medical Systems, Andover, MA, US) with 4-field box 3D plans or 9 field intensity modulation radiotherapy (IMRT) plans. EBRT plans were delivered with a total dose of 45Gy delivered in 25 fractions (1.8Gy per daily fraction). BT treatment boost were planned on Eclipse treatment planning system (Varian Medical Systems, Palo Alto, US). The BT boost plans were delivered immediately followed by the EBRT treatment with total dose of 28Gy in 4 fractions (7Gy per fraction and two fractions per week) or 30Gy in 5 fractions (6Gy per fraction and two fractions per week). The collected data include planning images and treatment plans. The patient was scheduled for follow-up examination every 2~ 3 months after treatment. Patients complaining of hematochezia were further examined by colonoscopy. Twelve patients scored as Grade ≥ 2 rectal toxicity per CTCAE v4 [19] were characterized as toxicity patients, and the other 30 Grade 0–1 patients were non-toxicity patients. To account for biologic effects of different fractionation schemes, both the rectum physical doses received in BT and EBRT were converted to EQD2 doses using a linear quadratic model [20] with an α/β ratio of 3 for dose summation [21, 22]. This retrospective study was approved by the institutional review board (IRB).

### Deformable dose accumulation and rectum unfolding

For all patients, the volume of rectum was defined as the total rectal wall segmented between the level of the ischial tuberosity and the rectosigmoid junction, with a length ranging from 6~ 9 cm in the patient cohort. The rectum surface meshes were generated using rectum contours via a particle-based surface meshing approach [23].

A previously developed local topology preserved non-rigid registration point matching algorithm (TOP-DIR) was employed for rectum surface registration [24]. Details of the TOP-DIR algorithm can be found in Additional file 1: Appendix A. We regarded the first BT fraction as the reference and registered the other BT fractions rectum surface to the reference fraction rectum surface to obtain the deformation vector fields (DVFs), which were used to deform and sum fractional BT rectal doses to yield cumulative BT rectal dose. Considering a homogenous dose distribution often covers the entire pelvic region in our EBRT treatment plan regimen, we assumed a homogenous EBRT dose in the pelvic region and added the EBRT dose to the BT cumulated dose without deformation to obtain the total EBRT+BT dose.

The EBRT+BT rectal dose was then flattened via a 3D-2D mapping to generate a 2D RSDM. The 3D-2D rectal dose mapping is detailed in Additional file 1: Appendix B.

### Dosimetric features extraction

**Fig. 1:**
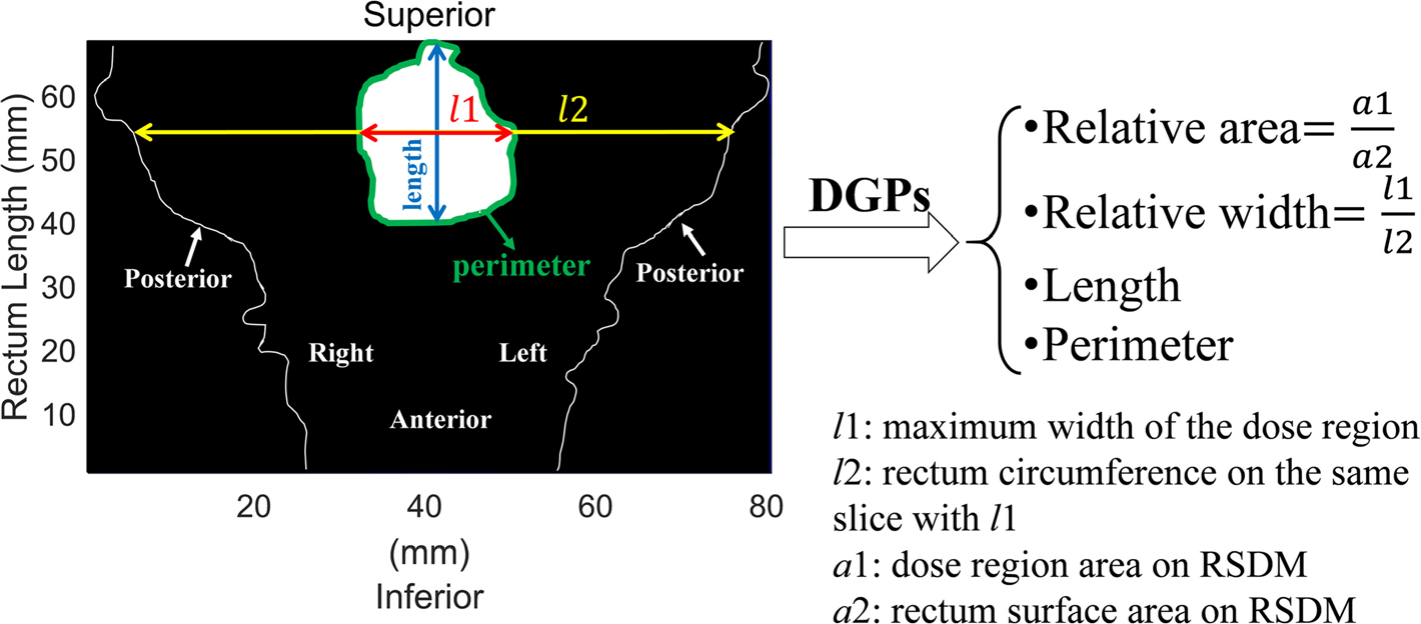
Example of DGPs extracted from the RSDM at a certain dose level

Three types of dosimetric features, DVPs, texture feature and DGPs were extracted. The DVPs (21 in total) were D*x*-cc (minimum dose in the most exposed *x*-cm^3^ volume, *x* ∈ [0.1,10] with 0.5cm^3^ intervals) calculated from the 3D EBRT+BT rectal dose. The texture features (43 in total) were extracted from the RSDM, including 3 first-order gray level statistical global features, 9 Gy level co-occurrence matrix (GLCM) texture features, 13 Gy level run-length matrix (GLRLM) texture features, 13 Gy level size zone matrix (GLSZM) texture features, and 5 neighborhood gray-tone difference matrix (NGTDM) texture features [25]. The DGPs (224 in total) were computed from the RSDM at various dose levels, ranging from 45Gy to 100Gy with 1Gy interval, including 1) the relative area (%) of the dose region with respect to the area of rectum surface in the RSDM; 2) the perimeter (mm) of the dose region; 3) the relative width (%), the ratio between the maximum width of the dose region with respect to the rectum circumference on the corresponding CT slice; and 4) the length (mm) of the dose region. The DGPs are illustrated in Fig. 1.

### Toxicity prediction scheme

We employed the support vector machine (SVM) [26] based method as our prediction scheme. A sequential forward feature selection (SFS) algorithm [27] was used to select a subset of features with best SVM prediction from the feature set (detailed in Additional file 1: Appendix C). We refer the above predictive scheme as *SVM-SFS* hereafter in this paper.

Considering the unbalanced training patient cohort where the toxicity group constitutes only a relative small portion of the dataset in this study, a synthetic minority over-sampling technique (SMOTE) [28] was used. The SMOTE balances the training dataset by over-sampling the minority class via introducing synthetic examples along the line segments joining *k* minority class nearest neighbors. This data balancing technic has been shown to be helpful for avoiding over-fitting and better model generalization [29–33]. In the five-fold cross validation in this study, data balancing was merely applied to the training dataset in each fold of validation, while the validation dataset was kept unchanged for its “purity”.

### Quantification and comparison

The rectum surface registration accuracy is measured by four similarity metrics [24, 34], including the Dice’s coefficient (DC), the percent error (PE), the mean vertex to vertex distance (VVD), and the Hausdorff distance (HD). Higher DC or lower PE, VVD, and HD indicate better results.

The predictive performance was quantified by the accuracy (ACC), sensitivity (SEN), specificity (SPE), and the area under the receiver operating characteristic (ROC) curve (AUC). An AUC of 0.5 is expected if a random prediction is performed. ACC, SEN and SPE are defined as: *ACC* = (*TP* + *TN*)/(*TP* + *FP* + *FN* + *TN*), SEN = *TP*/(*TP* + *FN*) and SPE = *TN*/(*TN* + *FP*), where *TP* is true positive, *TN* is true negative, *FP* is false positive and *FN* is false negative. The mean ACC, SEN, SPE and AUC via a repeated (100 times) 5-fold cross validation was reported.

For a comparison study, the conventional dose volume parameters D_0.1/1/2cm_^3^ calculated via the *“static-hotspot assumption”* (SA) approach [35] were used as a baseline (referred as SA-D_0.1/1/2cm_^3^). The SA-D_0.1/1/2cm_^3^ were compared to the features computed from the deformably summed EBRT+BT EQD2 dose on 3D rectum surfaces (DVPs) and flattened 2D RSDMs (texture features and DGPs). With many DVP and DGP parameters, it is possible to cause overfitting. To guard against overfitting, we extract representing features from DVPs and DGPs with 1) principle component analysis (PCA), and 2) statistical analyses (referred as **F**_**PCA**_ and **F**_**sta**_, respectively). The **F**_**PCA**_ were features in the PCA domain that calculated by performing the PCA on all the DVPs, texture features and DGPs with the first *n* principal components account for > 99% of the variance. The **F**_**sta**_ were computed by performing statistical analyses (Mann-Whitney U test with raw *p*-values reported) on each feature category of DVPs, texture features and DGPs between the toxicity and non-toxicity groups, to screen out those statistical significant features.

The prediction capabilities of SA-D_0.1/1/2cm_^3^, **F**_**PCA**_ and **F**_**sta**_ were compared by respectively feeding them into the SVM-SFS. The Z-test (*p*-values were adjusted by the Bonferroni correction) was used for ROC curves comparisons, and all the statistical analyses conducted in this study were considered significant if *p* < 0.05.

## Results

### Rectum DIR

**Fig. 2:**
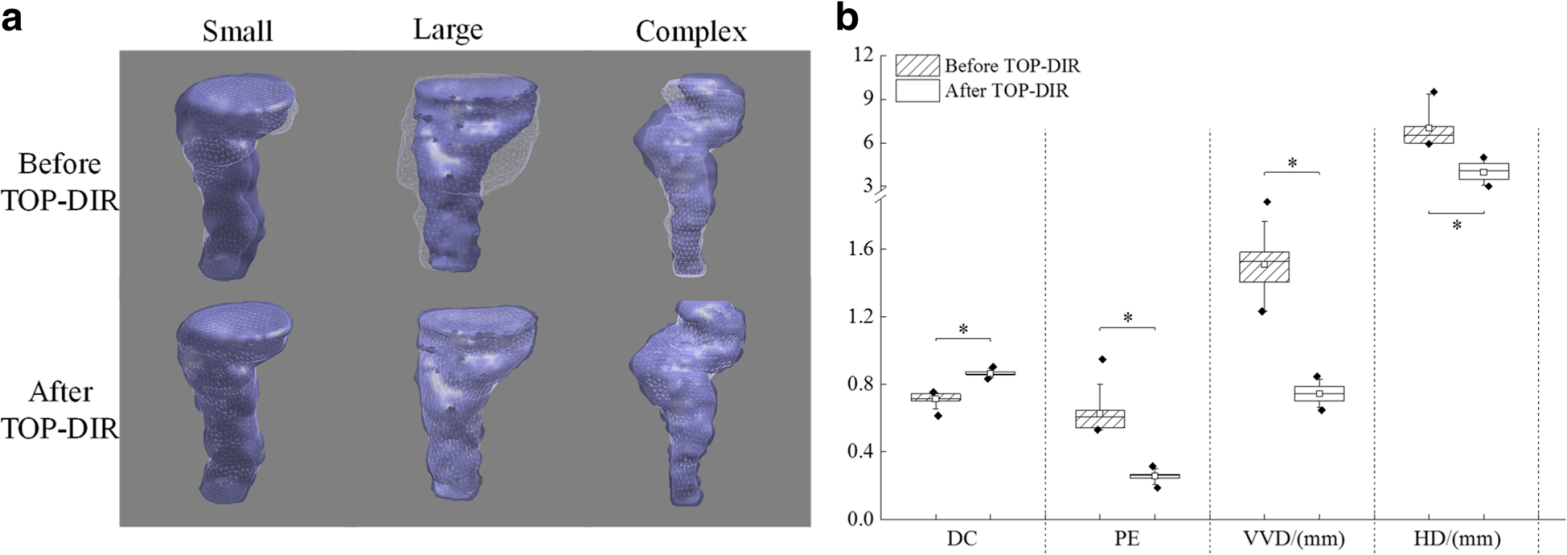
**a** Three example rectum TOP-DIRs with small, large and complex deformation. **b** Boxplots of DC, PE, VVD and HD over the patient groups before and after TOP-DIR. The boxes run from the 25th to 75th percentile; the two ends of the whiskers represent the 10 and 90% percentiles, the horizontal line and the square in the box represent the median and mean values, respectively. The diamonds represent outliers. Significant differences are marked with “*”

The TOP-DIR was demonstrated to be robust for different rectum DIR scenarios, as seen in three example cases (Fig. 2a) with small, large and complex deformation. For all the evaluated cases, 156 DIRs were performed, and the DC, PE, VVD and HD over the patient groups are depicted in Fig. 2b. Significant improvements were achieved after TOP-DIR point matching, with the median of DC increased from 0.71 to 0.86 (*p* < 0.001), the median of PE, VVD and HD decreased from 0.60, 1.53 mm and 6.52 mm to 0.26 (*p* < 0.001), 0.74 mm (*p* < 0.001) and 4.06 mm (*p* < 0.001), respectively.

### Representative features F_PCA_ and F_sta_

The computed ***F***_***PCA***_ were 10 representative PCA features that the first *n* = 10 principal components were used. While the **F**_**sta**_ were statistical significant (*p* < 0.05) features of DVPs, texture features and DGPs, which were identified via the Mann-Whitney U test. These significant features **F**_**sta**_ (73 in total) included: ① 13 DVPs from D_0.1cm_^3^ to D_6cc_; ② 6 texture features: *correlation* in GLCM, *long run high gray-level emphasis* (LRHGE) in GLRLM, *low gray-level zone emphasis* (LGZE), *high gray-level zone emphasis* (*HGZE*) and *small zone high gray-level emphasis*(*SZHGE*) in GLSZM, *complexity* in NGTDM; ③ 54 DGPs, including relative areas in dose levels of 55Gy~64Gy, 67Gy~68Gy, 87Gy~89Gy; perimeters in dose levels of 54Gy~65Gy, 87Gy~89Gy; relative widths in dose level of 87Gy~89Gy; and length in dose levels of 59Gy~60Gy, 63Gy~64Gy, 66Gy~73Gy, 79Gy~83Gy, 85Gy, 87Gy~89Gy.

### Prediction comparisons of SA-D_0.1/1/2cm_^3^, F_PCA_ and F_sta_

**Fig. 3:**
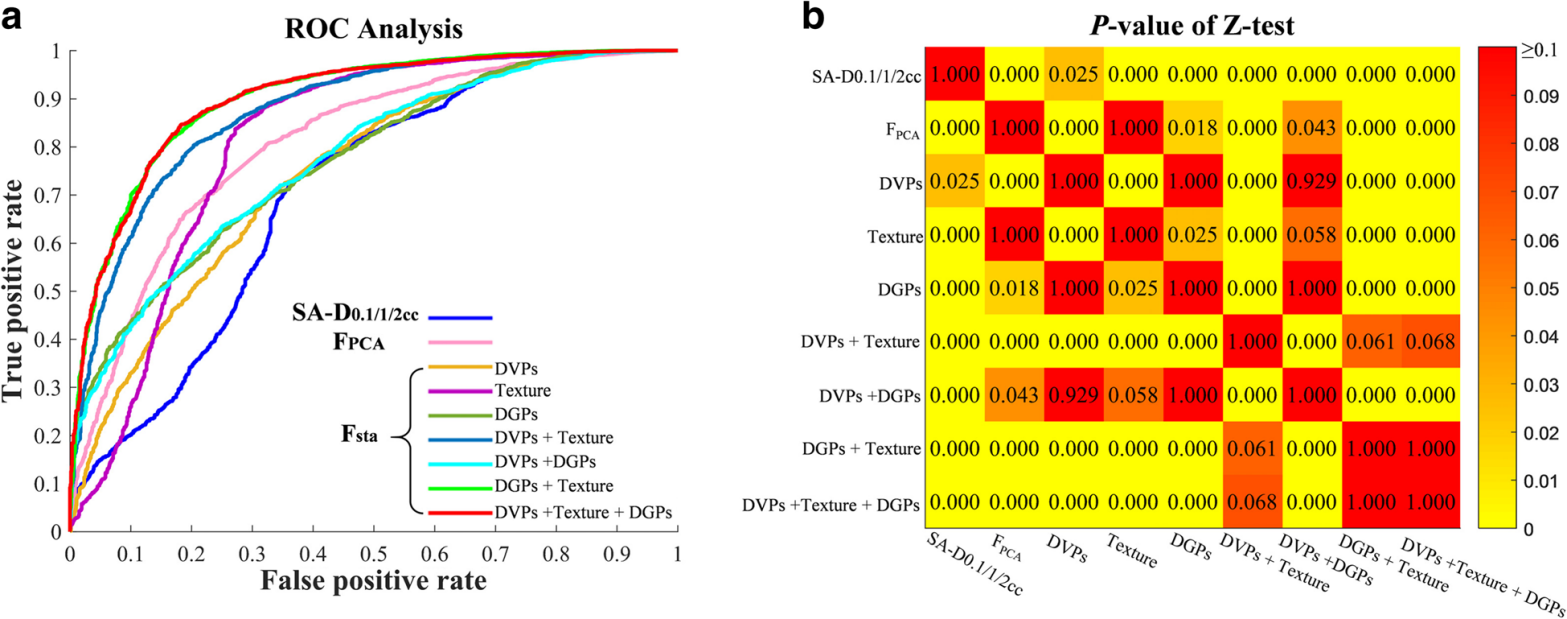
**a** ROC analysis for different significant features and their combinations via SVM-SFS. **b** ROC curves comparisons via Z-test (*p*-values were adjusted by the Bonferroni correction)

The prediction performance of SA-D_0.1/1/2cm_^3^**, F**_**PCA**_ and **F**_**sta**_ were listed in Table 1. For traditional SA-D_0.1/1/2cm_^3^, the prediction resulted in SEN 66.25%, SPE 66.73%, and AUC 0.71 (95% confidence interval [CI]: 0.68–0.72). For the **F**_**PCA**_, better prediction performances were observed when compared with SA-D_0.1/1/2cm_^3^ (*p* < 0.001), with SEN 74.75%, SPE 72.67%, and AUC 0.82 (95% CI: 0.75–0.85). While for **F**_**sta**_, we compared different combinations of the significant features of DVPs, texture features and DGPs in **F**_**sta**_. It is observed that using the DVPs alone in **F**_**sta**_ had only limited improvement when compared with SA-D_0.1/1/2cm_^3^ (*p* = 0.025). In contrast, using the texture features in **F**_**sta**_ or the DGPs in **F**_**sta**_ achieved better predictive performances than both the SA-D_0.1/1/2cm_^3^ and DVPs in **F**_**sta**_. The best predictive results were achieved by using the combinations of “DGPs + texture” or “DVPs + DGPs + texture” when compared with SA-D_0.1/1/2cm_^3^ (*p* < 0.001), with SEN 85.17%/84.75%, SPE 79.13%/79.87% and AUC 0.91 (95% CI: 0.85–0.92)/0.91 (95% CI: 0.87–0.93). The comparisons of SA-D_0.1/1/2cm_^3^**, F**_**PCA**_ and **F**_**sta**_ via SVM-SFS were depicted by the ROC analysis in Fig. 3.

**Table 1:** SVM-SFS prediction on different features

Features		SEN	SPE	ACC	AUC (95% CI)
SA-D_0.1/1/2cm_^3^		66.25%	66.73%	66.55%	0.71 (0.68–0.72)
F_PCA_		74.75%	72.67%	73.22%	0.82 (0.75–0.85)
F_sta_	DVPs	63.42%	73.20%	70.37%	0.76 (0.69–0.80)
	Texture	75.50%	73.23%	73.86%	0.82 (0.75–0.86)
	DGPs	60.42%	74.53%	70.46%	0.79 (0.72–0.81)
	DVPs + Texture	81.00%	78.60%	79.36%	0.88 (0.84–0.91)
	DVPs + DGPs	61.92%	73.83%	70.40%	0.79 (0.72–0.82)
	DGPs + Texture	85.17%	79.13%	80.84%	0.91 (0.85–0.92)
	DVPs + Texture + DGPs	**84.75%**	**79.87%**	**81.32%**	**0.91 (0.87–0.93)**

### Top ranked features statistics in F_sta_

**Fig. 4:**
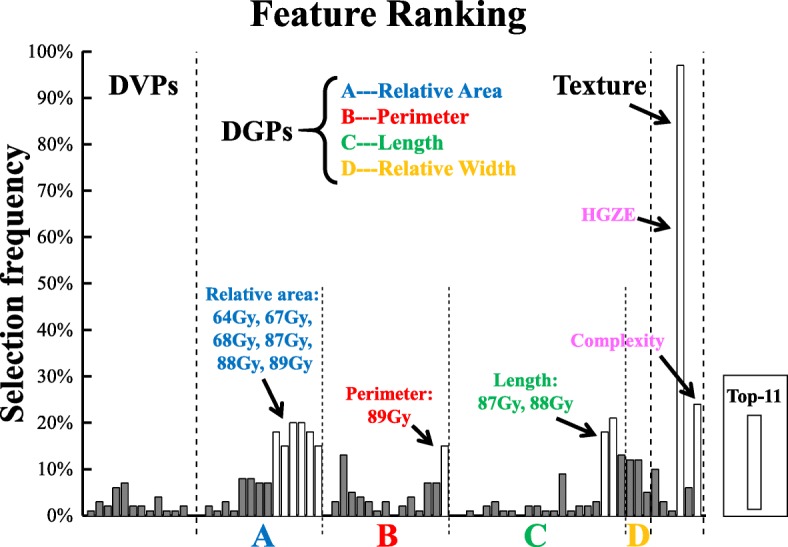
Feature ranking via SVM-SFS (repeated 100 times) on feature combinations of “DVPs + Texture + DGPs” from F_sta_

By utilizing all the 73 significant features of **F**_**sta**_, the SVM-SFS model was repeated 100 times and the features were ranked according to their frequencies of being selected. The feature selection frequency distributions are shown in Fig. 4. The top-10 features included relative areas in dose levels of 64 Gy, 67Gy, 68Gy, 87Gy~89Gy, perimeters in dose levels of 89Gy, length in dose levels of 87Gy and 88Gy, and two texture features: HGZE and complexity. No DVPs were ranked as the top-10 features.

Statitics of the top-10 features between the toxicity and non-toxicity groups are depicted in Table 2. For DGPs, the relative area in dose levels of 64Gy, 67Gy, 68Gy was significantly larger in the toxicity group (*p* = 0.034, 0.049 and 0.045, respectively). For the dose levels of 87Gy, 88Gy and 89Gy (with median = 0 for both toxicity and non-toxicity groups), differences in distributions for the relative area were observed (*p* = 0.023, 0.023 and 0.023, respectively). Similarly, the perimeter in dose levels of 89Gy and the length in dose levels of 87Gy and 88Gy (all with median = 0 for both toxicity and non-toxicity groups) have statistically significant differences in distributions between the two groups (*p* = 0.023, *p* = 0.016 and 0.023, respectively). For texture features, the median (interquartile range, IQR) of HGZE were 317.81(51.19) vs. 376.63(37.70) (*p* = 0.0007), and the complexity were 130.71(9.04) vs. 119.77(10.18) (*p* = 0.0087) between the two groups.

**Table 2:** Statistical analysis of the top 10 features in **F**_**sta**_ between the toxicity and non-toxicity groups

		DGPs										Texture		
		Relative area (%)							Perimeter (mm)	Length (mm)				
		64Gy	67Gy	68Gy	87Gy	88Gy	89Gy		89Gy	87Gy	88Gy		HGZE	Complexity
Median(IQR)	Toxi	25.26 (15.57)	19.07 (20.57)	16.87 (20.22)	0 (0.3)	0 (0.12)	0 (0.05)		0 (9.77)	0 (3.5)	0 (1.75)		317.81 (51.19)	130.71 (9.04)
	Non-toxi	16.87 (16.59)	12.26 (18.29)	9.48 (16.28)	0 (0)	0 (0)	0 (0)		0 (0)	0 (0)	0 (0)		376.63 (37.70)	119.77 (10.18)
P-Value		0.034	0.049	0.045	0.023	0.023	0.023		0.023	0.016	0.023		0.001	0.009

## Discussion

An effective rectal toxicity prediction scheme is essential for guiding radiation treatment planning. D_0.1/1/2cm_^3^ are recommended by the GEC-ESTRO guidelines [36] for rectum dose monitoring, however, their predictive capabilities for rectal toxicity are still under investigation. Other studies reported that the D_5cc_ may be a more reliable estimate than other dose volume parameters to predict risks of rectosigmoid mucosal changes and late rectal complications [37, 38]. All these studies essentially used DVPs to predict rectal toxicity. The findings in current work align with the previous studies, e.g., statistically significant differences were observed in DVPs ranging between D_0.1cm_^3^~D_6cc_. However, better prediction was accomplished by utilizing all the significant DVPs in **F**_**sta**_ when compared with merely using the SA-D_0.1/1/2cm_^3^ (Table 1).

In this study, we have compared two approaches, i.e., the PCA analysis and the statistical analysis, in extracting representative features for feeding the SVM-SFS prediction scheme. The merit of performing PCA is to reduce the number and correlation of the potential features by converting the features into a set of values of linearly uncorrelated variables (**F**_**PCA**_ in this study). However, these converted values in the PCA domain carry no physical meanings, it is therefore difficult to interpret the prediction why the **F**_**PCA**_ features are responsible for yielding corresponding prediction result. On the other hand, the statistical analysis approach reserves the physical meanings of the features by statistically pre-screening the significant features (**F**_**sta**_ in this study), and the comparison evaluations also showed superior performance if all the features in **F**_**sta**_ were used for prediction, when compared with **F**_**PCA.**_

Recently, researchers started to investigate the prediction model with spatial dose information. For instance, Buettner et al. presented a late rectal toxicity method based on the parameterized representation of the 3D rectal dose [39]. Lee et al. proposed a metric based on both surface dose and distance to predict incidence of the rectal bleeding in prostate cancer patients treated with radical radiotherapy [40]. Drean et al. identified rectal subregions at risk of rectal bleeding by performing voxel-wise analysis on the rectal dose distribution [7]. In this study, we took advantage of the hollow structure of the rectum and flattened the 3D rectal dose to 2D RSDM to establish dose map toxicity prediction scheme. Though the RSDM neglects the doses in the rectum thickness direction, it preserves spatial dose information. The texture features and the DGPs, which are crafted to capture spatial dose distribution characteristics from the 2D RSDM, are able to provide more geometric and positional dosimetric information. Pioneer studies have shown potential correlations of spatial dose characteristics with rectal toxicity. For example, Drean et al. reported that the rectal subregions at risk of rectal bleeding are primarily located in the subprostatic anterior hemi-rectum and upper part of the anal canal [7]. Kim et al. found substantial correlation between rectal toxicity and percent rectal circumference at certain dose levels. Similarly, in this study, we have seen texture features and the geometric dosimetric features had better predictive power than the DVPs. These results hint us that rectum’s response to dose might be dose-spatial dependent. As shown in Table 2 and Fig. 4, geometric feature such as the relative area, the perimeter and the length were found to be associated with rectal toxicity. The toxicity group tended to have larger dose coverage on the high dose region (64, 67 and 68Gy). This finding was in agreement with previous studies that rectal bleeding was significantly correlated with high-dose metrics [25, 41–43]. In addition, although only four DGPs were investigated in this work, other DGPs which were explored in previous studies also indicated associations with rectal toxicity. For instance, Buettner et al. investigated the eccentricity of the fitted ellipse of the dose region and found associations of the eccentricity with loose stools [5, 39, 44]. Moulton et al. also reported that compactness, circularity and confinement to the ellipse fits were correlated with rectal bleeding [18]. Incorporating these spatial features, which are crafted to depict the dose coverage and the shape of dose distribution, into the current model may potentially improve the predictive performance. Besides, two texture features, i.e., the HGZE and Complexity, were statistically different between the two groups, where the toxicity group has lower HGZE value (the Median(IQR) 317.81(51.19) vs. 376.63(37.70), *p* = 0.0007) but higher Complexity value (the Median(IQR) 130.71(9.04) vs. 119.77(10.18), *p* = 0.0087). However, how these texture features impact on rectal toxicity is still unclear. We applied the texture features on the RSDM with the intention to describe localized dosimetric patterns on the RSDM which are usually difficult to be noticed by human eyes. Yet, the drawbacks of the texture features are their deficiency of physical implications on interpreting correlations. Moreover, feature stability, e.g., whether the selected texture feature will change if different patient cohort size are used, is still an exploratory issue [45–47]. Since current work is a pioneer feasibility study of applying texture feature analysis on the deformably accumulative rectum surface dose map, more in-depth investigations on a larger patient cohort is still required in the future.

In this study, the generated 2D RSDM reserves the physical length of the rectum in both the superior-inferior direction and the circumferential direction on each slice (see Additional file 1: Appendix B). This was to ensure that the geometric features (e.g., area, perimeter, length, etc.) extracted from the RSDM would carry physical implications to signify the scale of dose delivered on rectum surface. Note that the drawback for reserving the physical dimension of the rectum is that the inter-patient variations of the rectum size could possibly influence/mask the significances of differences of the extracted geometric features between the two groups, especially given a small patient cohort. But the rectum size tends to be a random number across patients and therefore its impact would decrease and be minor in a larger patient cohort.

In addition, reporting accurate accumulated dose over the entire treatment course is a nontrivial task because of the substantial inter-fractional rectum deformation exists in the BT treatments. In this work, a previously developed TOP-DIR algorithm was used, although accurate geometric registration accuracy had been achieved and validated on a porcine bladder phantom (~ 2 mm), further phantom studies are still needed to justify its effectiveness in rectum registration, and the dosimetric errors in the subsequent dose summation step also need to be monitored.

In this study, we added the EBRT dose to the accumulated BT dose without deformation. The reasons are twofold: firstly, a homogenous dose distribution (hot spot < 107%) often covers the entire pelvic region in our EBRT treatment plan regimen. Often large portion of rectum are within treatment fields, especially for 3D plans. Only a very small inferior portion of the rectum is outside of the large pelvis treatment fields and dose variation across rectum is often within 15%. With this relative homogenous dose in a large region across the pelvis, we could assume rectum receiving a consistent and homogenous dose in EBRT as planned. In this study, the EBRT plans were generated with 4-field box 3D plans or 9 field IMRT plans. Theoretically, these two techniques on a same patient would result in different EBRT dose distributions due to dose conformity and hence different accumulated dose on RSDM. It is therefore more appropriate to investigate the extracted features for each technique. However, it is impractical to implement in current study since only a small patient sample was available. The influence of these two EBRT techniques on the stability of the extracted features still needs further investigations on a larger patient cohort.

On the other hand, DIR between EBRT and BT CTs is challenging because of the clinical use of the intracavitary applicator in BT. Registering the BT CT image with applicator to the EBRT CT image without applicator (or vice versa) is difficult, if not impossible, since the point-to-point correspondence assumption is usually violated in most DIR algorithms. Consequently, the dosimetric uncertainties via EBRT-BT DIR might be possibly even larger than that summed without deformation. There are several reported attempts to address this issue [48, 49], for example, Berendsen et al. [48] proposed a DIR with penalty term that minimizes the volume of the missing structure for cervical MR images with and without applicator. Vasquez Osorio et al. [49] validated a structure-wise registration with vector field integration to map the largely deformed anatomies between EBRT and BT. However, the EBRT-BT DIR needs to be treated prudently, and these novel methods need comprehensive validations before they can be confidently applied in a clinical setting. Adding EBRT to BT without deformation is therefore a reasonable approximation without knowing the uncertainties brought by the EBRT-BT DIR.

The choice of prediction models and feature selection strategies may also affect the predictive performance. We used the SVM-SFS scheme because it is the most common method to construct a predictive model with simultaneously feature selection. Though satisfactory performances have been achieved, other predictive models (e.g., random forest classifier) or feature selection methods (e.g., clonal selection algorithm) can provide even better predictive accuracy [50, 51].

For screening of the representative feature F_sta_, the unadjusted *p*-values were used for statistical analysis, however, the current findings will probably change if the *p*-values were corrected for multiple testing. In fact, *p*-value adjustment is restrictive to application with many tests and applying it in the context of RSDM analysis is still controversial [18, 52]. Since the physical length of the rectum was reserved on the RSDM in this study, the resolutions of the RSDMs were essentially patient specific. Multiple testing might not be applicable for this scenario where the resolution of the RSDM is fixed for each patient. Even though *p*-values corrections have been reported in other similar investigations using RSDM for rectal toxicity studies, however, the adjusted *p*-values did not demonstrate clear trends across regions on the RSDM where only limited and isolated regions of significance were found after applying multiple testing correction in RSDM analysis [18, 52, 53]. Furthermore, reporting the raw *p*-values is an exploratory study of finding predictive factors correlated to rectal toxicity without the risk of missing important factors which might be discarded if found to be insignificant after *p*-value adjustment [18].

One limitation of current study is that the patient cohort was small. Thus, the number of extracted features was larger than the patient sample size. To reduce the chance of getting over-fitting, a statistical analysis was performed to screen out the significant features before feeding into the predictive model. This guarantees a more robust feature ranking in the subsequent feature selection step in SVM-SFS. But note that a more effective way to observe overfitting is to separate the patient cohort into three datasets, i.e., one for training, one for validation and hyper-parameter tuning and one for testing. However, it was impossible to effectively separate our samples into three datasets, and cross validation was therefore our secondary option for model performance observation in a small patient cohort. But overfitting might also occur in the cross-validation space attributed to other factors such as the quantity of features considered, the selection of model hyper-parameters, etc., therefore, larger patient data is a key for evaluating model stability and generalization capability.

Another limitation of the study is that our study is purely on dosimetric parameter without consider clinical factors. Multivariable modeling of radiotherapy outcomes has been conducted by El Napa et al. [54]. We will further include clinical factors in our near future studies.

## Conclusions

In summary, we have proposed and validated a rectum toxicity prediction method based on an accurate point registration and machine learning for cervical cancer radiotherapy. The extensive experimental results have demonstrated the feasibility of the proposed scheme for rectal toxicity prediction, rendering it a potential tool for clinical OARs dose control and complication prediction.

## Additional file

Additional file 1: Appendix A. TOP-DIR algorithm. Appendix B. 3D-2D rectum surface dose mapping. Appendix C. Sequential forward feature selection (SFS) algorithm. (DOCX 155 kb)

## Abbreviations

ACC: accuracy
AUC: area under the receiver operating characteristic curve
BT: brachytherapy
DC: the Dice’s coefficient
DGPs: dose geometric parameters
DVFs: deformation vector fields
DVH: dose volume histogram
DVPs: dose volume parameters
EBRT: external beam radiotherapy
FN: false negative
FP: false positive
F_PCA_: representative PCA features extracted from DVPs, textures and DGPs
F_sta_: representative (statistically significant) features screened out from DVPs, textures and DGPs
GLCM: gray level co-occurrence matrix
GLRLM: gray level run-length matrix
GLSZM: gray level size zone matrix
HD: the Hausdorff distance
HGZE: high gray-level zone emphasis
IRB: institutional review board
LGZE: low gray-level zone emphasis
LRHGE: long run high gray-level emphasis
NGTDM: neighborhood gray-tone difference matrix
OARs: organs at risk
PCA: principle component analysis
PE: the percent error
RBF: radial basis function
ROC curve: receiver operating characteristic curve
RSDM: rectum surface dose map
SA: static-hotspot assumption
SA-D_0.1/1/2cm_^3^: dose volume parameters D_0.1/1/2cm_^3^ calculated using the “static-hotspot assumption” addition method
SEN: sensitivity
SFS: sequential forward feature selection
SMOTE: synthetic minority over-sampling technique
SPE: specificity
SVM: support vector machine
SZHGE: small zone high gray-level emphasis
TN: true negative
TOP-DIR: topology preserved point matching-deformable image registration
TP: true positive
VVD: the mean vertex to vertex distance
