# Supplementary material for: Investigating rectal toxicity associated dosimetric features with deformable accumulated rectal surface dose maps for cervical cancer radiotherapy
Source: Radiat Oncol. 2018 Jul 6;13:125. doi: 10.1186/s13014-018-1068-0 (PMC6035458; doi:10.1186/s13014-018-1068-0)
Supplement: Supplementary file 1 — Appendix A. TOP-DIR algorithm. Appendix B. 3D-2D rectum surface dose mapping. Appendix C. Sequential forward feature selection (SFS) algorithm. (DOCX 155 kb) [file 13014_2018_1068_MOESM1_ESM.docx]

**Additional file 1**

**Appendix A. TOP-DIR algorithm**

The TOP-DIR [[1](#_ENREF_1)] is a thin plate spline robust point matching (TPS-RPM) algorithm [[2](#_ENREF_2)] based point matching algorithm. Specifically, given the moving point sets $\boldsymbol{V}=\left\{ {\vec{\boldsymbol{v}}}_{i}={(v}_{i}^{x},v_{i}^{y},v_{i}^{z})|i=1,2,\ldots,K \right\}$ and the static point sets $\boldsymbol{X}=\left\{ {{\vec{\boldsymbol{x}}}_{j}=(x}_{j}^{x},x_{j}^{y},x_{j}^{z})|j=1,\cdots,N \right\}$ in $\mathcal{R}^{3}$, the TPS-RPM finds the optimal correspondence $\boldsymbol{M}$ and a smooth non-rigid transformation $f$ between $\boldsymbol{X}$ and $\boldsymbol{V}$ by minimizing$\arg\min_{\boldsymbol{M},f} E(\boldsymbol{M},f)=\sum_{i=1}^{K+1} \sum_{j=1}^{N+1} m_{ij}\left\| {\vec{\boldsymbol{x}}}_{j}-f({\vec{\boldsymbol{v}}}_{i}) \right\|^{2}+\lambda\left\| Lf \right\|^{2}+T\sum_{i=1}^{K} \sum_{j=1}^{N} m_{ij}\log m_{ij}+T_{0}\sum_{j=1}^{N} m_{N+1,j}\log m_{N+1,j}+T_{0}\sum_{i=1}^{K} m_{i,K+1}\log m_{i,K+1}$, where $L$ is a thin-plate splines (TPS) smoothness regularization operator, $\boldsymbol{M}$ is a fuzzy correspondence matrix to characterize the correspondence between $\boldsymbol{X}$ and $\boldsymbol{V}$. To preserve local topological information, a correspondence matrix $P_{ij}$ based on $m_{ij}$ is formulated as:$P_{ij}=\frac{m_{ij}S_{ij}}{\sum_{k=1}^{N} m_{ik}S_{ik}}$, $S_{ij}=\sum_{s\in N_{V}^{i}} \sum_{t\in N_{X}^{j}} R_{ij}(s,t)m_{st}$, where $R_{ij}(s,t)$ measures the compatibility between the neighboring point pair $(i,s)$ in $\boldsymbol{V}$ and $(j,t)$ in $\boldsymbol{X}$ that $R_{ij}\left( s,t \right)=\alpha\beta$,$\alpha\left( i,s;j,t \right)=1-\left| \frac{\left( d\left( i,s \right)-d\left( j,t \right) \right)}{\max_{m\in N_{V}^{i},n\in N_{X}^{j}} \left( d\left( i,m \right)-d\left( j,n \right) \right)} \right|$ and $\beta\left( i,s;j,t \right)=\left( 1-d\left( i,s \right)/\max_{m\in N_{V}^{i}} (d\left( i,m \right)) \right)\left( 1-d\left( j,t \right)/\max_{n\in N_{X}^{j}} (d\left( j,n \right)) \right)$, where $d\left( \cdot,\cdot\right)$ is the spatial distance between two points. $P_{ij}$ is incorporated into the TPS-RPM framework and used to substitute the original correspondence matrix $m_{ij}$ in each iteration step.

**Appendix B. 3D-2D rectum surface dose mapping**

The 3D-2D rectum surface dose mapping procedure began with visiting every rectum contour point in a clockwise direction starting from the most posterior position on each CT slice. The corresponding doses point equally spaced along each contour were recorded and then ordered as a row vector. By traversing all the slices from superior to inferior, a 2D dose matrix (i.e., the rectum surface dose map (RSDM)) was obtained with rows representing doses delivered on each contour point on every CT slice and then was converted to physical length based on the image pixel size. The 2D RSDMs have 1mm×1mm resolution for all cases but with patient specific image sizes that depend on the number of rectum contour points on each slice and the number of CT slices. The rectum unfolding is illustrated in Figure S1.

|  |
| --- |
| **Figure S1** Example of unfolding the 3D rectum surface dose (left) to a 2D rectum surface dose map (right). |

**Appendix C.** **Sequential forward feature selection (SFS) algorithm**

The goal of feature selection is to select the most important features from the original feature set to construct an effective classifier. The process of feature selection can be mathematically formulated as: given a feature set $F=(f_{1},f_{2},\ldots, f_{n})$, a classifier is constructed with a criterion function $J(\cdot)$ of the selected features. Feature selection is to select a subset $S$ in $F$ such that $J\left( S \right)>J(T)$, where $T$ is all possible subset of $F$. The sequential forward selection (SFS) starts with an empty set $X$=0, and the classification accuracy from the *K*-fold (*K*=5 in this study) cross validation was used as the criterion function $J$. Select the first feature that has the highest $J$ among all features. Then select the feature among all unselected features together with the selected features that gives the highest $J$. Repeat the above process until the desired accuracy is reached.

**References**

1. Haibin C, Zichun Z, Yuliang L, Arnold P, Brian H, Kevin A, Xin Z, Linghong Z, Xuejun G: **A non-rigid point matching method with local topology preservation for accurate bladder dose summation in high dose rate cervical brachytherapy.** *Physics in Medicine and Biology* 2016, **61:**1217.

2. Chui H, Rangarajan A: **A new point matching algorithm for non-rigid registration.** *Comput Vis Image Underst* 2003, **89:**114-141.
